# 2-(1*H*-Benzimidazol-1-yl)-1-(2-fur­yl)ethanone *O*-isopropyl­oxime

**DOI:** 10.1107/S1600536809022302

**Published:** 2009-06-17

**Authors:** Özden Özel Güven, Taner Erdoğan, Simon J. Coles, Tuncer Hökelek

**Affiliations:** a Department of Chemistry, Zonguldak Karaelmas University, 67100 Zonguldak, Turkey; b Department of Chemistry, Southampton University, Southampton SO17 1BJ, England; c Department of Physics, Hacettepe University, 06800 Beytepe, Ankara, Turkey

## Abstract

In the mol­ecule of the title compound, C_16_H_17_N_3_O_2_, the planar benzimidazole ring system [maximum deviation = 0.015 (2) Å] is oriented at a dihedral angle of 72.17 (4)° with respect to the furan ring. An intra­molecular C—H⋯O inter­action results in the formation of a six-membered ring having an envelope conformation. In the crystal structure, inter­molecular C—H⋯N inter­actions link the mol­ecules into centrosymmetric *R*_2_^2^(18) dimers.

## Related literature

For general background to oximes and oxime ethers, including their biological activity, see: Baji *et al.* (1995 ▶); Bhandari *et al.* (2009 ▶); Emami *et al.* (2002 ▶, 2004 ▶); Milanese *et al.* (2007 ▶); Polak (1982 ▶); Poretta *et al.* (1993 ▶); Ramalingan *et al.* (2006 ▶); Rosello *et al.* (2002 ▶). For related structures, see: Özel Güven *et al.* (2007*a* ▶,*b* ▶, 2009 ▶). For ring-motifs, see: Bernstein *et al.* (1995 ▶).
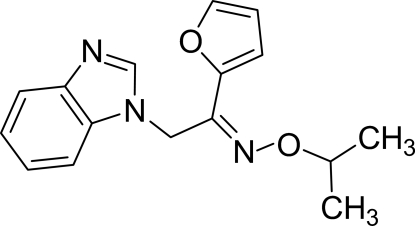


## Experimental

### 

#### Crystal data

C_16_H_17_N_3_O_2_
*M* *_r_* = 283.33
Monoclinic, 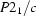

*a* = 8.4290 (2) Å
*b* = 17.7606 (3) Å
*c* = 10.6017 (2) Å
β = 111.882 (1)°
*V* = 1472.77 (5) Å^3^
*Z* = 4
Mo *K*α radiation
μ = 0.09 mm^−1^
*T* = 120 K
0.40 × 0.20 × 0.20 mm

#### Data collection

Bruker–Nonius KappaCCD diffractometer
Absorption correction: multi-scan (*SADABS*; Sheldrick, 2007 ▶) *T*_min_ = 0.966, *T*_max_ = 0.979
20597 measured reflections
3356 independent reflections
2803 reflections with *I* > 2σ(*I*)
*R*_int_ = 0.035

#### Refinement

*R*[*F* ^2^ > 2σ(*F* ^2^)] = 0.042
*wR*(*F* ^2^) = 0.111
*S* = 1.12
3356 reflections
259 parameters
All H-atom parameters refined
Δρ_max_ = 0.27 e Å^−3^
Δρ_min_ = −0.28 e Å^−3^

### 

Data collection: *COLLECT* (Hooft, 1998 ▶); cell refinement: *DENZO* (Otwinowski & Minor, 1997 ▶) and *COLLECT*; data reduction: *DENZO* and *COLLECT*; program(s) used to solve structure: *SHELXS97* (Sheldrick, 2008 ▶); program(s) used to refine structure: *SHELXL97* (Sheldrick, 2008 ▶); molecular graphics: *ORTEP-3 for Windows* (Farrugia, 1997 ▶); software used to prepare material for publication: *WinGX* (Farrugia, 1999 ▶) and *PLATON* (Spek, 2009 ▶).

## Supplementary Material

Crystal structure: contains datablocks I, global. DOI: 10.1107/S1600536809022302/im2123sup1.cif

Structure factors: contains datablocks I. DOI: 10.1107/S1600536809022302/im2123Isup2.hkl

Additional supplementary materials:  crystallographic information; 3D view; checkCIF report

## Figures and Tables

**Table 1:** Hydrogen-bond geometry (Å, °)

*D*—H⋯*A*	*D*—H	H⋯*A*	*D*⋯*A*	*D*—H⋯*A*
C11—H11⋯O2	0.98 (2)	2.32 (2)	2.772 (2)	107 (1)
C13—H13⋯N2^i^	0.96 (2)	2.37 (2)	3.286 (2)	159 (1)

Symmetry code: (i) 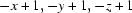.

**Fig. 1.**
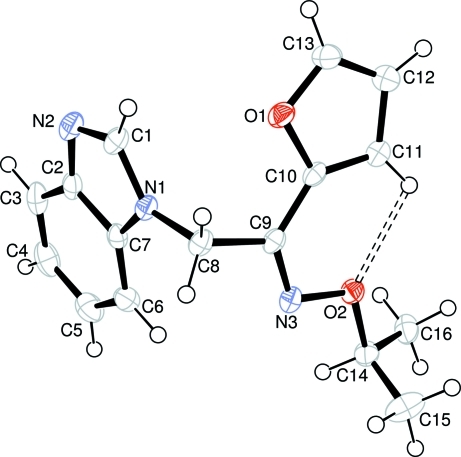
The molecular structure of the title molecule with the atom-numbering scheme. Displacement ellipsoids are drawn at the 50% probability level. Hydrogen bond is shown as dashed line.

**Fig. 2.**
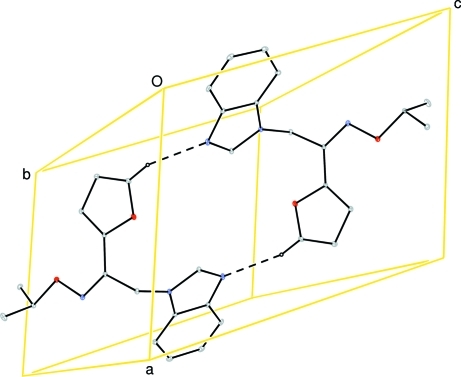
A partial packing diagram of the title compound. Hydrogen bonds are shown as dashed lines. H atoms not involved in hydrogen bonding have been omitted for clarity.

## supplementary crystallographic information

### Comment

Oximes and oxime ethers show very important antifungal and antibacterial activities. Oxiconazole is a well established drug for treatment of many mycotic infections, having an oxime group (Polak, 1982). Several compounds containing an oxime or an oxime ether function have been reported to exhibit antimicrobial activity (Poretta *et al.*, 1993; Baji *et al.*, 1995; Rosello *et al.*, 2002; Emami *et al.*, 2002; Emami *et al.*, 2004; Ramalingan *et al.*, 2006; Milanese *et al.*, 2007; Bhandari *et al.*, 2009). In our earlier studies, we reported X-ray structures of benzimidazole substituted oxiconazole derivatives (Özel Güven *et al.*, 2007*a*; 2007*b*; 2009). Now, we report herein the crystal structure of the title alkyl oxime ether.

In the molecule of the title compound (Fig. 1), the bond lengths and angles are generally within normal ranges. The planar benzimidazole ring system [with a maximum deviation of 0.015 (2) Å for atom C5] is oriented with respect to the furan ring at a dihedral angle of 72.17 (4)°. Atoms C8 and C9 are -0.037 (1) and 0.008 (1) Å away from the furan ring plane, respectively, while atom C8 is at a distance of -0.008 (1) Å to the benzimidazole ring plane. So, they are coplanar with the adjacent rings. The N1—C1—N2 [114.1 (1)°], N2—C2—C7 [110.2 (1)°], C2—C7—C6 [122.8 (1)°], C3—C4—C5 [121.7 (1)°] and C4—C5—C6 [121.8 (1)°] bond angles are enlarged, while C5—C6—C7 [116.2 (1)°] and C2—C3—C4 [117.5 (1)°] bond angles are narrowed. An Intramolecular C—H···O interaction (Table 1) results in the formation of a six-membered ring, (O2/N3/C9—C11/H11), having envelope conformation with atom H11 displaced by -0.126 (15) Å from the plane of the other ring atoms.

In the crystal structure, intermolecular C—H···N interactions (Table 1) link the molecules into centrosymmetric dimers exhibiting *R*_2_^2^(18) ring motifs (Bernstein *et al.*, 1995) (Fig. 2).

### Experimental

The title compound was synthesized by the reaction of 2-(1*H*-benzimidazol-1-yl)-1-(furan-2-yl)ethanone oxime obtained from 2-(1*H*-benzimidazol-1-yl)-1-(furan-2-yl)ethanone (Özel Güven *et al.*, 2007*b*) with iso-propyl bromide and NaH. To a solution of 2-(1*H*-benzimidazol-1-yl)-1-(furan-2-yl)ethanone oxime (400 mg, 1.658 mmol) in DMF (5 ml) was added NaH (66 mg, 1.658 mmol) in small fractions. Then, iso-propyl bromide (204 mg, 1.658 mmol) was added dropwise. The mixture was stirred at room temperature for 3 h and the excess of hydride was decomposed with a small amount of methanol. After evaporation to dryness under reduced pressure, the crude residue was suspended with water and extracted with methylene chloride. The organic layer was dried over anhydrous sodium sulfate and then evaporated to dryness. The crude residue was purified by chromatography on a silica-gel column using chloroform and recrystallized from ethyl acetate to obtain yellow crystals (yield; 126 mg, 27%).

### Refinement

All H atoms were located from difference Fourier syntheses and refined isotropically [C—H = 0.948 (17)–1.057 (18) Å, *U*_iso_(H) = 0.022 (3)–0.061 (6) Å^2^].

### Crystal data

**Table:**

C_16_H_17_N_3_O_2_	*F*(000) = 600
*M**_r_* = 283.33	*D*_x_ = 1.278 Mg m^−^^3^
Monoclinic, *P*2_1_/*c*	Mo *K*α radiation, λ = 0.71073 Å
Hall symbol: -P 2ybc	Cell parameters from 3444 reflections
*a* = 8.4290 (2) Å	θ = 2.9–27.5°
*b* = 17.7606 (3) Å	µ = 0.09 mm^−^^1^
*c* = 10.6017 (2) Å	*T* = 120 K
β = 111.882 (1)°	Plate, yellow
*V* = 1472.77 (5) Å^3^	0.40 × 0.20 × 0.20 mm
*Z* = 4	

### Data collection

**Table:**

Bruker–Nonius KappaCCD diffractometer	3356 independent reflections
Radiation source: fine-focus sealed tube	2803 reflections with *I* > 2σ(*I*)
graphite	*R*_int_ = 0.035
Detector resolution: 9.091 pixels mm^-1^	θ_max_ = 27.5°, θ_min_ = 3.1°
φ and ω scans	*h* = −10→10
Absorption correction: multi-scan (*SADABS*; Sheldrick, 2007)	*k* = −23→23
*T*_min_ = 0.966, *T*_max_ = 0.979	*l* = −13→12
20597 measured reflections	

### Refinement

**Table:**

Refinement on *F*^2^	Secondary atom site location: difference Fourier map
Least-squares matrix: full	Hydrogen site location: inferred from neighbouring sites
*R*[*F*^2^ > 2σ(*F*^2^)] = 0.042	All H-atom parameters refined
*wR*(*F*^2^) = 0.111	*w* = 1/[σ^2^(*F*_o_^2^) + (0.0577*P*)^2^ + 0.2982*P*] where *P* = (*F*_o_^2^ + 2*F*_c_^2^)/3
*S* = 1.12	(Δ/σ)_max_ < 0.001
3356 reflections	Δρ_max_ = 0.27 e Å^−^^3^
259 parameters	Δρ_min_ = −0.28 e Å^−^^3^
0 restraints	Extinction correction: *SHELXL97* (Sheldrick, 2008), Fc^*^=kFc[1+0.001xFc^2^λ^3^/sin(2θ)]^-1/4^
Primary atom site location: structure-invariant direct methods	Extinction coefficient: 0.091 (6)

### Special details

**Table:**

Geometry. All e.s.d.'s (except the e.s.d. in the dihedral angle between two l.s. planes) are estimated using the full covariance matrix. The cell e.s.d.'s are taken into account individually in the estimation of e.s.d.'s in distances, angles and torsion angles; correlations between e.s.d.'s in cell parameters are only used when they are defined by crystal symmetry. An approximate (isotropic) treatment of cell e.s.d.'s is used for estimating e.s.d.'s involving l.s. planes.
Refinement. Refinement of *F*^2^ against ALL reflections. The weighted *R*-factor *wR* and goodness of fit *S* are based on *F*^2^, conventional *R*-factors *R* are based on *F*, with *F* set to zero for negative *F*^2^. The threshold expression of *F*^2^ > σ(*F*^2^) is used only for calculating *R*-factors(gt) *etc*. and is not relevant to the choice of reflections for refinement. *R*-factors based on *F*^2^ are statistically about twice as large as those based on *F*, and *R*- factors based on ALL data will be even larger.

### Fractional atomic coordinates and isotropic or equivalent isotropic displacement parameters (Å^2^)

**Table:**

	*x*	*y*	*z*	*U*_iso_*/*U*_eq_	
O1	0.58682 (11)	0.47550 (5)	0.80555 (8)	0.0252 (2)	
O2	0.41827 (11)	0.33276 (5)	1.02341 (8)	0.0247 (2)	
N1	0.19899 (13)	0.45752 (5)	0.64047 (9)	0.0205 (2)	
N2	0.18669 (14)	0.44880 (6)	0.42568 (10)	0.0278 (3)	
N3	0.30106 (13)	0.38505 (6)	0.94150 (10)	0.0226 (2)	
C1	0.25496 (17)	0.48323 (7)	0.54285 (12)	0.0244 (3)	
H1	0.3367 (18)	0.5250 (8)	0.5619 (14)	0.025 (3)*	
C2	0.07716 (15)	0.39595 (7)	0.44673 (12)	0.0241 (3)	
C3	−0.03026 (17)	0.34334 (8)	0.35695 (13)	0.0318 (3)	
H3	−0.032 (2)	0.3408 (9)	0.2646 (17)	0.038 (4)*	
C4	−0.12897 (19)	0.29830 (9)	0.40499 (15)	0.0386 (4)	
H4	−0.203 (2)	0.2612 (11)	0.3450 (18)	0.051 (5)*	
C5	−0.12247 (18)	0.30444 (8)	0.53863 (15)	0.0360 (3)	
H5	−0.196 (2)	0.2724 (10)	0.5696 (17)	0.045 (5)*	
C6	−0.01575 (16)	0.35542 (7)	0.62975 (14)	0.0282 (3)	
H6	−0.0111 (19)	0.3597 (8)	0.7232 (16)	0.031 (4)*	
C7	0.08317 (14)	0.40074 (6)	0.58065 (11)	0.0212 (3)	
C8	0.25028 (16)	0.48361 (7)	0.78092 (12)	0.0222 (3)	
H81	0.3035 (17)	0.5328 (8)	0.7868 (13)	0.022 (3)*	
H82	0.1471 (19)	0.4885 (8)	0.8028 (14)	0.026 (4)*	
C9	0.37140 (15)	0.42870 (6)	0.87967 (11)	0.0197 (3)	
C10	0.54860 (15)	0.42703 (6)	0.89174 (11)	0.0198 (3)	
C11	0.69233 (16)	0.38863 (7)	0.96787 (12)	0.0236 (3)	
H11	0.6964 (19)	0.3507 (9)	1.0364 (15)	0.030 (4)*	
C12	0.82605 (17)	0.41455 (7)	0.92724 (13)	0.0289 (3)	
H12	0.941 (2)	0.3979 (9)	0.9589 (17)	0.040 (4)*	
C13	0.75635 (17)	0.46619 (8)	0.82951 (13)	0.0296 (3)	
H13	0.799 (2)	0.4959 (9)	0.7728 (16)	0.037 (4)*	
C14	0.33484 (16)	0.28212 (7)	1.08724 (13)	0.0265 (3)	
H14	0.219 (2)	0.2716 (9)	1.0207 (15)	0.031 (4)*	
C15	0.3264 (3)	0.31843 (10)	1.21285 (18)	0.0463 (4)	
H151	0.446 (3)	0.3321 (12)	1.281 (2)	0.061 (6)*	
H152	0.254 (3)	0.3634 (12)	1.189 (2)	0.060 (6)*	
H153	0.272 (3)	0.2848 (11)	1.2585 (19)	0.060 (5)*	
C16	0.44161 (18)	0.21125 (8)	1.11657 (14)	0.0316 (3)	
H161	0.446 (2)	0.1863 (10)	1.0273 (18)	0.048 (5)*	
H162	0.398 (2)	0.1768 (10)	1.1676 (18)	0.048 (5)*	
H163	0.559 (2)	0.2239 (9)	1.1768 (17)	0.040 (4)*	

### Atomic displacement parameters (Å^2^)

**Table:**

	*U*^11^	*U*^22^	*U*^33^	*U*^12^	*U*^13^	*U*^23^
O1	0.0290 (5)	0.0255 (4)	0.0222 (4)	−0.0023 (3)	0.0109 (4)	0.0052 (3)
O2	0.0236 (5)	0.0258 (4)	0.0235 (4)	−0.0009 (3)	0.0075 (3)	0.0090 (3)
N1	0.0244 (5)	0.0197 (5)	0.0171 (5)	0.0029 (4)	0.0074 (4)	0.0016 (4)
N2	0.0345 (6)	0.0279 (5)	0.0204 (5)	0.0050 (4)	0.0098 (4)	0.0049 (4)
N3	0.0253 (5)	0.0231 (5)	0.0179 (5)	0.0015 (4)	0.0063 (4)	0.0020 (4)
C1	0.0310 (7)	0.0213 (6)	0.0212 (6)	0.0025 (5)	0.0100 (5)	0.0049 (4)
C2	0.0236 (6)	0.0251 (6)	0.0200 (6)	0.0078 (5)	0.0038 (5)	0.0027 (4)
C3	0.0300 (7)	0.0332 (7)	0.0227 (6)	0.0077 (6)	−0.0012 (5)	−0.0023 (5)
C4	0.0270 (7)	0.0369 (7)	0.0388 (8)	−0.0018 (6)	−0.0029 (6)	−0.0080 (6)
C5	0.0258 (7)	0.0353 (7)	0.0435 (8)	−0.0040 (6)	0.0088 (6)	0.0002 (6)
C6	0.0240 (6)	0.0307 (6)	0.0310 (7)	0.0018 (5)	0.0115 (5)	0.0023 (5)
C7	0.0189 (6)	0.0212 (5)	0.0209 (6)	0.0057 (4)	0.0043 (4)	0.0007 (4)
C8	0.0288 (7)	0.0197 (6)	0.0185 (6)	0.0030 (5)	0.0092 (5)	−0.0010 (4)
C9	0.0259 (6)	0.0182 (5)	0.0146 (5)	−0.0004 (4)	0.0069 (4)	−0.0026 (4)
C10	0.0272 (6)	0.0175 (5)	0.0149 (5)	−0.0032 (4)	0.0082 (4)	−0.0014 (4)
C11	0.0274 (6)	0.0226 (6)	0.0203 (6)	−0.0002 (5)	0.0082 (5)	0.0004 (4)
C12	0.0260 (7)	0.0323 (7)	0.0289 (7)	−0.0009 (5)	0.0110 (5)	0.0000 (5)
C13	0.0282 (7)	0.0340 (7)	0.0297 (7)	−0.0046 (5)	0.0144 (5)	0.0010 (5)
C14	0.0251 (6)	0.0283 (6)	0.0252 (6)	−0.0067 (5)	0.0082 (5)	0.0061 (5)
C15	0.0648 (12)	0.0454 (9)	0.0409 (9)	−0.0056 (9)	0.0340 (9)	0.0029 (7)
C16	0.0288 (7)	0.0292 (7)	0.0306 (7)	−0.0061 (5)	0.0039 (6)	0.0094 (5)

### Geometric parameters (Å, °)

**Table:**

O1—C10	1.3782 (13)	C6—H6	0.980 (15)
O1—C13	1.3650 (16)	C8—C9	1.5142 (16)
O2—N3	1.3976 (12)	C8—H81	0.973 (14)
O2—C14	1.4547 (14)	C8—H82	0.984 (15)
N1—C1	1.3661 (15)	C9—C10	1.4511 (17)
N1—C7	1.3823 (15)	C10—C11	1.3621 (17)
N1—C8	1.4625 (15)	C11—C12	1.4247 (18)
N2—C1	1.3096 (16)	C11—H11	0.981 (15)
N2—C2	1.3917 (17)	C12—H12	0.948 (17)
N3—C9	1.2929 (15)	C13—C12	1.3440 (19)
C1—H1	0.981 (15)	C13—H13	0.964 (17)
C2—C3	1.3988 (18)	C14—C15	1.505 (2)
C2—C7	1.4043 (17)	C14—H14	0.988 (16)
C3—C4	1.381 (2)	C15—H151	1.02 (2)
C3—H3	0.974 (16)	C15—H152	0.98 (2)
C4—H4	0.966 (19)	C15—H153	0.98 (2)
C5—C4	1.402 (2)	C16—C14	1.5107 (19)
C5—H5	0.983 (17)	C16—H161	1.057 (18)
C6—C5	1.3836 (19)	C16—H163	0.982 (17)
C6—C7	1.3919 (18)	C16—H162	0.973 (19)
			
C13—O1—C10	106.74 (9)	N3—C9—C10	126.47 (10)
N3—O2—C14	110.34 (9)	N3—C9—C8	114.73 (11)
C1—N1—C7	106.29 (10)	C10—C9—C8	118.73 (10)
C1—N1—C8	127.66 (10)	C11—C10—O1	109.24 (10)
C7—N1—C8	126.05 (10)	C11—C10—C9	136.17 (11)
C1—N2—C2	104.24 (10)	O1—C10—C9	114.58 (10)
C9—N3—O2	111.22 (9)	C10—C11—C12	106.75 (11)
N2—C1—N1	114.13 (11)	C10—C11—H11	124.1 (9)
N2—C1—H1	125.1 (8)	C12—C11—H11	129.2 (9)
N1—C1—H1	120.7 (8)	C13—C12—C11	106.61 (12)
N2—C2—C3	129.89 (12)	C13—C12—H12	125.5 (10)
N2—C2—C7	110.19 (10)	C11—C12—H12	127.8 (10)
C3—C2—C7	119.91 (12)	C12—C13—O1	110.65 (11)
C4—C3—C2	117.53 (13)	C12—C13—H13	134.2 (10)
C4—C3—H3	123.9 (10)	O1—C13—H13	115.1 (9)
C2—C3—H3	118.6 (10)	O2—C14—C15	109.71 (11)
C3—C4—C5	121.71 (13)	O2—C14—C16	104.87 (10)
C3—C4—H4	118.9 (10)	C15—C14—C16	113.33 (12)
C5—C4—H4	119.4 (10)	O2—C14—H14	107.9 (9)
C6—C5—C4	121.81 (14)	C15—C14—H14	110.6 (9)
C6—C5—H5	118.2 (10)	C16—C14—H14	110.2 (9)
C4—C5—H5	120.0 (10)	C14—C15—H151	111.3 (11)
C5—C6—C7	116.20 (12)	C14—C15—H152	110.8 (12)
C5—C6—H6	121.9 (9)	H151—C15—H152	109.8 (17)
C7—C6—H6	121.9 (9)	C14—C15—H153	111.1 (11)
N1—C7—C6	132.02 (11)	H151—C15—H153	108.3 (15)
N1—C7—C2	105.14 (10)	H152—C15—H153	105.3 (16)
C6—C7—C2	122.83 (11)	C14—C16—H161	112.4 (9)
N1—C8—C9	111.54 (9)	C14—C16—H163	108.7 (10)
N1—C8—H82	108.4 (8)	H161—C16—H163	108.5 (14)
C9—C8—H82	108.8 (8)	C14—C16—H162	108.6 (11)
N1—C8—H81	107.7 (8)	H161—C16—H162	112.2 (14)
C9—C8—H81	111.0 (8)	H163—C16—H162	106.1 (14)
H82—C8—H81	109.4 (12)		
			
C13—O1—C10—C11	0.18 (12)	C7—C2—C3—C4	0.80 (18)
C13—O1—C10—C9	−179.51 (10)	N2—C2—C7—N1	−0.13 (13)
C10—O1—C13—C12	−0.41 (14)	C3—C2—C7—N1	−179.70 (10)
C14—O2—N3—C9	−177.47 (9)	N2—C2—C7—C6	178.84 (11)
N3—O2—C14—C15	−82.98 (13)	C3—C2—C7—C6	−0.73 (18)
N3—O2—C14—C16	155.00 (9)	C2—C3—C4—C5	−0.2 (2)
C7—N1—C1—N2	0.10 (14)	C6—C5—C4—C3	−0.6 (2)
C8—N1—C1—N2	179.73 (11)	C7—C6—C5—C4	0.7 (2)
C1—N1—C7—C6	−178.81 (12)	C5—C6—C7—N1	178.63 (12)
C8—N1—C7—C6	1.55 (19)	C5—C6—C7—C2	−0.03 (18)
C1—N1—C7—C2	0.03 (12)	N1—C8—C9—N3	−100.58 (12)
C8—N1—C7—C2	−179.62 (10)	N1—C8—C9—C10	76.58 (13)
C1—N1—C8—C9	−104.20 (13)	N3—C9—C10—C11	−5.0 (2)
C7—N1—C8—C9	75.38 (14)	C8—C9—C10—C11	178.23 (12)
C2—N2—C1—N1	−0.17 (14)	N3—C9—C10—O1	174.60 (10)
C1—N2—C2—C3	179.70 (12)	C8—C9—C10—O1	−2.19 (14)
C1—N2—C2—C7	0.18 (13)	O1—C10—C11—C12	0.10 (13)
O2—N3—C9—C10	−1.02 (15)	C9—C10—C11—C12	179.69 (12)
O2—N3—C9—C8	175.89 (9)	C10—C11—C12—C13	−0.34 (14)
N2—C2—C3—C4	−178.67 (12)	O1—C13—C12—C11	0.46 (15)

### Hydrogen-bond geometry (Å, °)

**Table:**

*D*—H···*A*	*D*—H	H···*A*	*D*···*A*	*D*—H···*A*
C11—H11···O2	0.98 (2)	2.32 (2)	2.772 (2)	107 (1)
C13—H13···N2^i^	0.96 (2)	2.37 (2)	3.286 (2)	159 (1)

Symmetry codes: (i) −*x*+1, −*y*+1, −*z*+1.
